# Exploring the potential of artificial intelligence in assessing the risk of gastric neoplastic lesions in patients with corpus atrophic gastritis

**DOI:** 10.1007/s10120-025-01679-7

**Published:** 2025-10-22

**Authors:** Emanuele Dilaghi, Edoardo Cesaroni, Irene Ligato, Matteo Silvestri, Giampaolo Liuzzi, Bruno Annibale, Stefano Lucidi, Gianluca Esposito, Marco Sciandrone

**Affiliations:** 1 Department of Medical-Surgical Sciences and Translational Medicine, Sant’Andrea Hospital, Sapienza University of Rome, Via di Grottarossa 1035, 00189 Rome, Italy; 2 Department of Computer, Control, and Management Engineering “Antonio Ruberti” – Sapienza University of Rome, Rome, Italy; 3 Department of Computer Science, Sapienza University of Rome, Rome, Italy

**Keywords:** Corpus atrophic gastritis, Gastric cancer, Artificial intelligence, Gastric neoplastic lesion, One-class support vector machine

## Abstract

**Background:**

Corpus atrophic gastritis (CAG) requires endoscopic-histological surveillance due to the risk of developing gastric neoplastic lesions (GNL). This study aimed to identify variables associated with GNL development at long-term follow-up using a Fisher score-based feature-ranking-approach coupled with a One-Class Support-Vector-Machine (SVM) model.

**Methods:**

A dataset containing 30 clinical, endoscopic, and histological variables from consecutive CAG patients (2001–2023) adhering to a surveillance-program was considered. GNL presence at the longest available follow-up was recorded. Gastric biopsies and histological evaluations followed the updated-Sydney-system. A Fisher score-based feature ranking method and a One-Class SVM were employed to select key variables linked to GNL development, and then validated with synthetically generated data.

**Results:**

Overall, 355 CAG patients were initially considered. Of these, 36 were excluded due to the presence of GNL at baseline gastroscopy, and 216 for missing data. Thus, a total of 103 patients were considered and grouped into: CAG patients with [22 patients (F 68.1%), median-age 68(35–83) years] and without GNL at follow-up [81 patients (F 72.8%) median-age 59(26–84) years]. After a median follow-up of 60(12–192) months, 13 epithelial GNL (gastric adenocarcinoma or high/low-grade dysplasia) and nine type-1 gastric-neuroendocrine-tumors (T1gNET) were recorded. Parietal-cell-antibodies and pepsinogen-I < 30 μg/l were associated with epithelial GNL and T1gNET. Antral inflammation and age > 60 were linked to epithelial GNL, while anti-thyroperoxidase-antibodies, smoking, and dyspeptic-symptoms were linked to T1gNET. Low-dose aspirin and *H. pylori* eradication therapy showed inverse associations with epithelial GNL and T1gNET, respectively.

**Conclusions:**

This is the first study in which an AI-model simultaneously considers clinical, endoscopic, and histological features from a dataset of CAG patients, showing the potential to identify variables associated with GNL development.

**Supplementary Information:**

The online version contains supplementary material available at 10.1007/s10120-025-01679-7.

## Introduction

Corpus atrophic gastritis (CAG) is a non-self-limiting chronic inflammatory condition affecting the corpus mucosa, characterized by the progressive decrease of the oxyntic glands, which may be replaced by pseudopyloric or intestinal metaplasia (IM) [1–3]. The subsequent reduced mass of specialized parietal cells leads to ipo- achlorhydria status, eventually resulting in micronutrient malabsorption and raising the risk of type 1 neuroendocrine tumor (T1gNET) and gastric cancer (GC) development [1–4].

Nowadays, the association between CAG and gastric cancer is debated [5–7]. In a retrospective single-center study of 275 CAG patients with a median follow-up of 5 years (range 1–17), annual incidence rates were 0.5% for GC, 0.6% for low-grade dysplasia, and 2.8% for T1gNET [7]. Age > 60 years, IM without pseudopyloric metaplasia, and pernicious anemia predicted GC or dysplasia, while pernicious anemia predicted T1gNET. Regular endoscopic-histologic follow-up is recommended by European MAPS II guidelines [8].

Artificial intelligence (AI), with its computational power and machine-learning algorithms like random forest and support vector machine (SVM), has gained significant attention in clinical practice [9]. The recent development of efficient hardware and computational power led to several AI models that could be applied in the clinical setting, endoscopy, radiology, and pathology to improve the diagnosis, treatment, and prognosis of many gastrointestinal conditions [10–15]. Despite the potential role of AI, the spread and use of this tool in clinical practice is still limited, as the real potential has yet to be fully explored, even in CAG.

This study aimed to assess the most impactful epidemiological, clinical, endoscopic, and/or histological variables associated with the development of epithelial lesions and T1gNETs over time using an SVM model.

## Methods

This article was drafted according to Standards for Reporting of Diagnostic Accuracy Studies guidelines to ensure the quality of reporting [16].

### Study population

We retrospectively analyzed a demographic, clinical, laboratory, endoscopic, and histological dataset from a cohort of consecutive patients diagnosed with CAG adhering to a clinical and an endoscopic-histological follow-up program. Outpatient visits were scheduled annually. Endoscopic follow-ups were scheduled every 4 years before 2011 and then every 3 years according to the European guidelines for the management of gastric precancerous conditions [8, 17, 18]. In case of new symptoms, worsening of known symptoms, or onset of anemia, a gastroscopy and/or outpatient visit were anticipated. The study population derived from a prospective historical cohort of patients with histological diagnosis of CAG between 2001 and 2023 according to internationally agreed criteria [1–4] at Sant’Andrea Hospital, Sapienza University of Rome. It is worth mentioning that, in this study, the term “corpus atrophic gastritis” was preferred over “autoimmune gastritis”. The histological diagnosis of CAG can be univocally established, whereas diagnostic criteria for autoimmune gastritis are not universally shared. Moreover, with traditional diagnostic tools, the etiology of this condition cannot always be unequivocally determined [19]. For the purposes of this study, CAG was defined as the histological presence of corpus atrophy assessed on gastric biopsies, with or without intestinal or pseudopyloric metaplasia, and in the absence of antral atrophy or IM.

Inclusion criteria were: age older than 18 years; diagnosis of CAG confirmed by histopathological assessment of gastric biopsies; completeness of gastric biopsy sampling protocol according to the updated Sydney system [20]; presence of at least an endoscopic-histological follow-up. Exclusion criteria were: history of esophagogastric malignancies; previous partial or total gastrectomy; patients who did not undergo follow-up gastroscopy. *Helicobacter pylori* (*H. pylori*) histologically positive patients were not excluded from the study, but all patients underwent eradication therapy at baseline [21]. The eventual ongoing proton pump inhibitor (PPI) therapy was stopped at least 2 weeks before gastroscopy; after CAG diagnosis, PPIs were withdrawn since their use could be questionable in such a hypochlorhydria condition [22, 23]. The observational period was defined as the time in months between the histologically confirmed diagnosis of CAG and the last available follow-up at the time of data analysis. If gastric neoplastic lesions (GNL) occurred during endoscopic-histological follow-up, this was the final follow-up considered. The incidence of GNL at the longest available follow-up was recorded. All patients provided informed consent. The study was conducted in adherence to the principles outlined in the Declaration of Helsinki.

### Endoscopic procedures

Expert endoscopists performed gastroscopies. All patients underwent pharyngeal anesthesia (xylocaine spray puffs) and conscious sedation (midazolam 3–5 mg). Gastroscopies were performed using white light, and since 2013, also using electronic chromoendoscopy. In case of suspicion of GNL, endoscopic characterization was first obtained, and then target biopsies were collected and sent in separate vials. In case of no suspected gastric lesions, biopsies were collected according to the updated Sydney system [20]. All biopsies were sent for histopathological evaluation in 2 separate vials, one containing the biopsies from the antrum and *incisura angularis* and the other from the corpus mucosa.

### Biopsies and histopathological assessment

Expert pathologists of the upper gastrointestinal tract performed the histopathological assessment. The histopathological report was redacted according to the criteria of the updated Sydney system [20]. CAG was defined as the decrease or the disappearance of the oxyntic glands that could be replaced by fibrosis or, most frequently, by pseudopyloric or IM. The presence of corpus atrophy was graded on a 4-grade scale: absence of replacement (score 0), replacement of mild degree (score 1), moderate degree (score 2), and severe degree (score 3). Corpus IM was defined as the substitution of the normal oxyntic glands with intestinalized glands [20]. The definition of GNL was based on the Padova international classification and World Health Organization guidelines [24, 25]: low-grade IEN, high-grade (HG) IEN, and GC. T1gNET was diagnosed when enterochromaffin-like cell nodular proliferation was > 500 mm in diameter [26]. Immunohistochemistry was performed using monoclonal antibodies against chromogranin A (Clone DAK-A3; Dako, Glostrup, Denmark) and Ki67 (Clone MIB1; Dako). Tumor grading was assessed according to the European Neuroendocrine Tumor Society grading system [27].

### Statistical analysis, variable selection, and model validation

The dataset included 30 variables for each patient, divided into demographic, clinical, laboratory, endoscopic, and histological data (Table 1). Statistical analysis was performed focusing on two distinct patient groups. The first group consisted of patients without GNL at both baseline and the longest available follow-up, while the second group included those who developed GNL during follow-up.

**Table 1:** The variables considered by the support vector machine model proposed

Variable	Type
Body mass index	Continuous
Hemoglobin level	Continuous
Mean corpuscular volume	Continuous
Ferritin level	Continuous
Vitamin B12 levels	Continuous
Homocysteine levels	Continuous
Observation interval between the diagnosis and the last available follow-up	Continuous
Age over 60	Binary
Gender	Binary
Active smoking	Binary
First degree of family history of gastric cancer	Binary
Presence of autoimmune comorbidities	Binary
Use of low-dose aspirin	Binary
Presence of dyspepsia at the baseline evaluation	Binary
Low pepsinogen I level < 30 μg/L	Binary
Parietal-cells antibodies	Binary
Antibodies against *Helicobacter pylori*	Binary
Anti-thyroperoxidase antibodies	Binary
Presence of iron deficiency anemia	Binary
Presence of pernicious anemia	Binary
Presence of *Helicobacter pylori* in the antrum	Binary
Presence of *Helicobacter pylori* in the corpus	Binary
Referred *Helicobacter pylori* eradication therapy prior to the CAG diagnosis	Binary
*Helicobacter pylori* eradication therapy prescribed at the CAG diagnosis	Binary
Presence of gastric neoplastic lesions at the last available follow-up	Binary
Corpus inflammation (mononuclear cell infiltration) level	Discrete (Scale from 0 to 3)
Antral inflammation (mononuclear cell infiltration) level	Discrete (Scale from 0 to 3)
Antral activity indicating active inflammation (neutrophilic inflammation)	Discrete (Scale from 0 to 3)
Corpus atrophy	Discrete (Scale from 0 to 3)
Corpus intestinal metaplasia	Discrete (Scale from 0 to 3)

*CAG* corpus atrophic gastritis

Taking into account that the dataset presented missing data, distinct strategies were adopted for data imputation:

- Continuous variables such as body mass index, hemoglobin, mean corpuscular volume, ferritin, vitamin B_12_, homocysteine, and observation interval were imputed using median values for lesion patients;
- Discrete variables **(**e.g., c-atrophy absent, mild, moderate, or severe**)** underwent imputation using the mode to maintain categorical integrity;
- In the group of patients with no GNL at baseline or follow-up, being the largest group, all patients with any missing data were excluded to leverage the robust dataset size.

Continuous and discrete variables were normalized using the Standard-Scaler method to ensure uniformity for subsequent analysis.

Three analyses were performed: the first contrasted patients with no GNL at baseline or follow-up with those presenting epithelial lesions; the second focused on patients with T1gNET, and the third grouped all patients with GNL and compared them to patients without GNL for a comprehensive assessment. In each analysis, imputation was performed using the median mode specific to the corresponding subgroup.

The statistical analysis was conducted using Python 3.12.1, with essential libraries such as NumPy for numerical operations, Pandas for data manipulation, and Scikit-learn for machine learning tasks. Additionally, Microsoft Excel was utilized for further data manipulation and visualization.

Following pre-processing, further analyses were conducted on the dataset to determine which variables were most impactful in developing lesions through time in patients with corpus gastric atrophy.

A generic patient “*i*” was represented by a vector${{x}^{\left(i\right)}\in R}^{29}$, where the components ${x}_{h}^{\left(i\right)}$ of the vector, for h = 1,..., 29, contained the values of the variables associated with the given patient. The thirtieth binary variable ${y}_{i}$ ∈ {−1, 1} indicated whether the patient “*i*” developed a lesion or not. Rather than developing predictive models, this research aimed to establish an optimal subset of variables that ensured effective separability between the two patient cohorts.

A two-step process was implemented in the analysis: first, a feature ranking method was applied to score the 29 features in decreasing order; then, taking into account this ordering, suitable subsets of variables were constructed, and a machine learning model assessed the goodness of each subset of variables. A Fisher Score-based method was employed as the feature ranking technique [28]. This method assigned a higher score to features with greater discriminant ability. Based on the resulting feature ranking, the One-Class SVM with a linear kernel [9, 29] was employed to evaluate the classification performance of various subsets of variables. The specific characteristics of the presented model enable the identification of variables crucial for evaluating CAG patients and recognizing those at increased risk of GNL. The One-Class SVM assigns + 1 to points in a defined region containing most training vectors and -1 to points outside. It separates vectors with a hyperplane, where coefficients indicate positive or negative correlations with lesion development.

A linear kernel was chosen to preserve the interpretability of the resulting model. This variant of SVM was especially effective in anomaly detection, making it well-suited for identifying rare diseases or unusual patterns in medical diagnostics. To select the optimal subset of variables, the performance of the One-Class SVM on the dataset was evaluated using the well-known leave-one-out cross-validation method. This approach facilitated the assessment of model performance by calculating average accuracy. Specifically, sensitivity and specificity were calculated for each validation run. The average values of these metrics were then used to determine the best subset of variables.

To further validate the model beyond leave-one-out cross-validation, an additional validation phase was performed using synthetically generated data. Specifically, we employed Conditional Tabular Generative Adversarial Network (CTGAN) [30], a generative model designed to synthesize tabular data while preserving the statistical distributions and dependencies of the original dataset. The model was implemented using the Synthetic Data Vault (SDV) Python library.

Three synthetic datasets were generated, each corresponding to one of the target clinical groups: patients without GNL, patients who developed epithelial GNL, and patients who developed T1gNET. For each group, the number of synthetic samples was matched to the size of the corresponding original training dataset. Model hyperparameters for each CTGAN instance were optimized through grid search to maximize the average of two evaluation scores, Support Vector Classifier (SVC) Detection and Logistic Detection, which assess the ability of an SVC and a logistic regression model, respectively, to distinguish real from synthetic data based on the area under the receiver operating characteristic curve. Values close to 1 indicate high-quality synthetic data.

The synthetic datasets were subsequently used to test the generalizability of the One-Class SVM classifiers trained on real data. Performance was evaluated using only the variable subsets selected in the main analysis, and sensitivity and specificity were computed. A detailed description of the evaluation metrics used for assessing the quality of synthetic data is available in the SDV documentation [31].

## Results

**Fig. 1:**
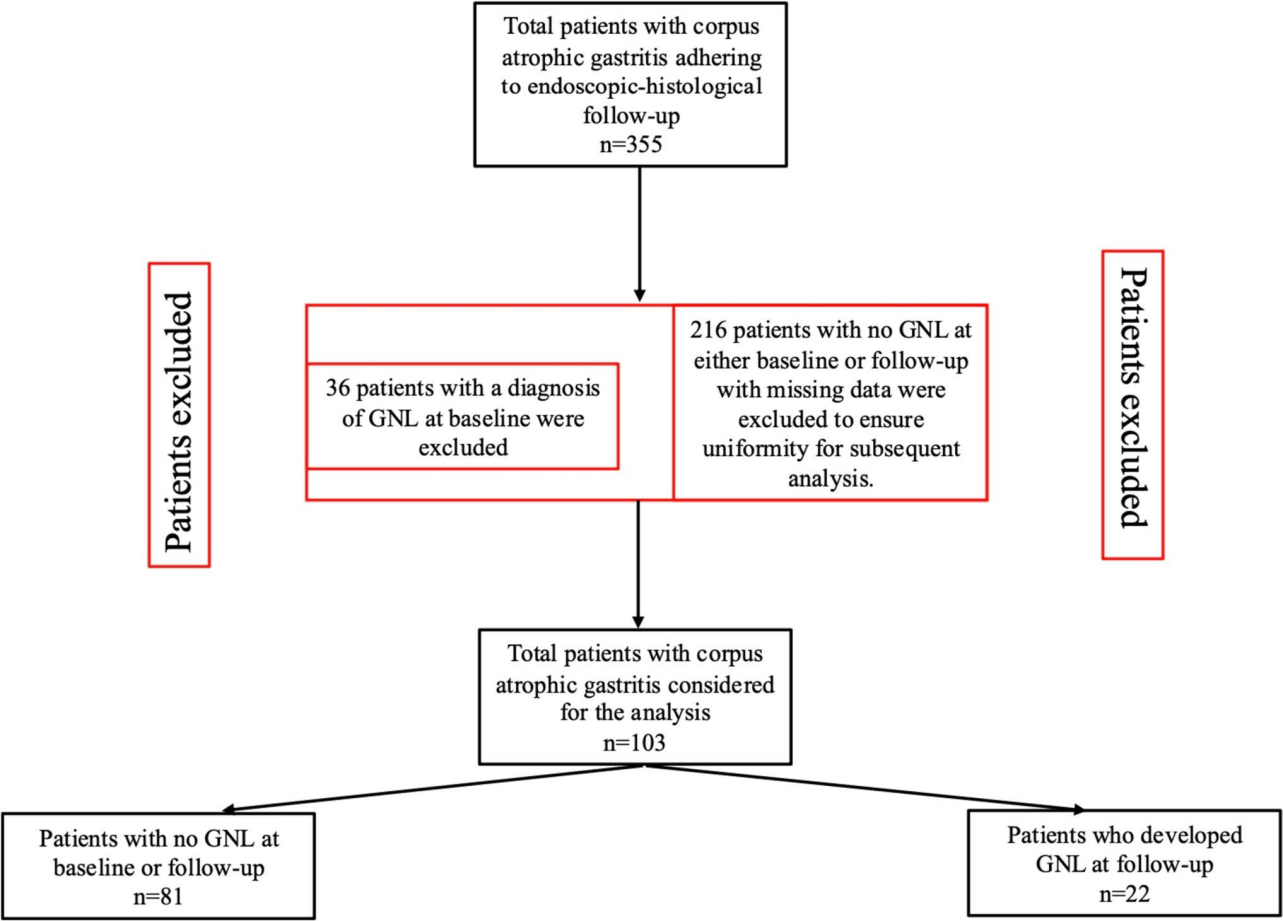
Flowchart of the study population of patients with corpus atrophic gastritis included in the analysis for identifying variables associated with gastric neoplastic lesion development using a One-Class Support Vector Machine. *GNL* gastric neoplastic lesions

A total of 355 patients with CAG [253 (71.3%) female, median age 61 (range 23–86) years] was initially considered. After pre-processing, 36 patients were excluded due to the presence of GNL at baseline gastroscopy, and 216 patients for missing data. The group of patients with no GNL at baseline or follow-up included 81 patients [59 (72.8%) female, median age 59 (range 26–84) years, median follow-up 60 (range 12–192) months], and the group of patients who developed GNL at follow-up included 22 patients [15 (68.1%) female, median age 68 (range 35–83) years, median follow-up 54 (range 8–144) months)]. The flowchart of the study population is reported in Fig. 1.

Among patients who developed GNL at follow-up, 13 patients were found to have an epithelial lesion, namely GC or HG/low-grade dysplasia, and 9 patients developed T1gNET.

The Fisher Score-based analysis revealed that subsets containing more than ten variables showed a significant decline in average sensitivity (Table 2). For this reason, the ten top-ranked features identified by the Fisher Score method were selected for further analysis. Subsets of features with cardinalities of 6 to 9 were created by selectively removing variables, and their classification performance was assessed. Ultimately, 385 subsets were tested, with those achieving average sensitivity and specificity above 80% selected for their balance in identifying true positives while minimizing false positives.

**Table 2:** Classification performance of progressively larger subsets of features, selected according to the Fisher score ranking

Subset cardinality	Added feature	*Se*^*—*^	*Sp*^*—*^
2	Pepsinogen I < 30 μg/L and anti-thyroperoxidase antibodies	0.23	0.53
3	Parietal-cells antibodies	0.77	0.77
4	*Helicobacter pylori* eradication therapy prescribed at CAG diagnosis	0.86	0.67
5	Age over 60	0.64	0.88
6	Mean corpuscular volume	0.91	0.77
7	Active smoking	0.91	0.79
8	Hemoglobin level	0.77	0.9
9	Antral inflammation	0.73	0.89
10	Use of low-dose of aspirin	0.68	0.91
11	Antibodies against *Helicobacter pylori*	0.68	0.92
12	Presence of *Helicobacter pylori* in the corpus	0.68	0.92
13	Corpus inflammation	0.55	0.92
14	Homocysteine levels	0.55	0.93
15	Presence of pernicious anemia	0.55	0.93
16	Presence of iron deficiency anemia	0.5	0.93
17	Presence of *Helicobacter pylori* in the antrum	0.55	0.92
18	Presence of dyspepsia at the baseline evaluation	0.5	0.94
19	Ferritin level	0.45	0.94
20	First degree of family history of gastric cancer	0.45	0.94
21	Referred *Helicobacter pylori* eradication therapy prior to the CAG diagnosis	0.45	0.94
22	Antral activity	0.45	0.94
23	Corpus atrophy	0.45	0.94
24	Corpus intestinal metaplasia	0.41	0.96
25	Gender	0.45	0.96

*Se* sensibility, *Sp* specificity

### Variables identified by the SVM-based model as predictors of epithelial GNL

The SVM-based model identified a subset of variables associated with the development of epithelial GNL during follow-up: age above 60 years, antral inflammation, presence of parietal-cells antibodies (PCA), and pepsinogen I levels below 30 µg/l. An inverse association was observed with the ongoing therapy with low-dose aspirin (Table 3). The coefficient associated with the variable anti-thyroperoxidase antibodies is zero, indicating that this variable can be excluded from the analysis without affecting the performance. These features achieved an average sensitivity of 92.3% and a specificity of 77.9%.

**Table 3:** Variables and respective coefficients associated with epithelial gastric neoplastic lesion development

Variable	Hyperplane’s coefficient
Anti-thyroperoxidase antibodies	0.00
Age over 60	0.27
Use of low-dose of aspirin	−0.46
Parietal-cells antibodies	0.60
Pepsinogen I < 30 μg/L	0.62
Antral inflammation	0.28

### Variables identified by the SVM-based model as predictors of T1gNET

The variables associated with T1gNET were the presence of PCA, pepsinogen I levels below 30 µg/l, presence of anti-thyroperoxidase antibodies, presence of dyspepsia at the time of CAG diagnosis, and active smoking status. An inverse association was found between T1gNET development and *H. pylori* eradication therapy at the time of CAG diagnosis (Table 4). These results achieved an average sensitivity of 77.8% and specificity of 92.6%.

**Table 4:** Variables and respective coefficients associated with the type 1 gastric neuroendocrine tumor development

Variable	Hyperplane’s coefficient
Anti-thyroperoxidase antibodies	0.36
Presence of dyspepsia at the baseline evaluation	0.37
Active smoking	0.35
Parietal-cells antibodies	0.41
*Helicobacter pylori* eradication therapy prescribed at CAG diagnosis	−0.68
Pepsinogen I < 30 μg/L	0.43

### Variables identified by the SVM-based model as predictors of overall GNL development

The SVM-based model was also applied to the overall outcome of GNL development, without distinguishing between epithelial GNL and T1gNET. The analysis identified a subset of variables associated with GNL occurrence, including the presence of anti-thyroperoxidase antibodies, age > 60 years, presence of PCA, pepsinogen I levels < 30 µg/L, antral inflammation, and active smoking. Inverse associations were observed with *H. pylo*ri eradication therapy at the time of CAG diagnosis and with hemoglobin levels (Table 5).

**Table 5:** Variables and respective coefficients associated with gastric neoplastic lesion development

Variable	Hyperplane’s coefficient
Anti-thyroperoxidase antibodies	0.27
Age over 60	0.64
*Helicobacter pylori* eradication therapy prescribed at CAG diagnosis	−0.58
Parietal-cells antibodies	1.00
Pepsinogen I < 30 μg/L	1.03
Antral inflammation	0.34
Active smoking	0.17
Hemoglobin level	−0.37

Specifically, this feature subset recorded an average sensitivity of 81.8% and a specificity of 89.1%.

### Validation on synthetic data

To further assess the robustness of the proposed model, its performance was tested on synthetic datasets generated for each clinical subgroup. The quality of these synthetic datasets was confirmed by high evaluation scores across multiple statistical metrics, indicating a close resemblance to the real data (Supplementary Table 1).

The One-Class SVM classifiers, trained on real data and evaluated on synthetic test sets, demonstrated high discriminative performance. For epithelial GNL, the model achieved a sensitivity of 92.3% and specificity of 86.4%; for T1gNET, sensitivity was 88.9% and specificity 92.6% (Supplementary Table 2). These findings provide further support for the discriminative power and generalizability of the selected feature subsets.

## Discussion

To the best of our knowledge, this is the first study to apply a Fisher-score ranking method with a One-Class SVM model to identify variables linked to GNL development in CAG patients. This approach highlights key risk factors distinguishing patients who develop GNL from those who do not. In particular, the choice of the machine learning model, i.e., One-Class SVM, a novelty detection model, was suggested because the dataset was strongly imbalanced. The machine learning model’s strong performance with selected variables confirms the validity of the methodology and results. The selected variables should be considered collectively, not individually. It is important to note that the SVM-based model is a classification tool, and the coefficients assigned to each variable reflect their contribution to the model’s discriminative performance. These coefficients do not imply causal relationships but rather indicate statistical associations. The specific characteristics of the presented model enable the identification of variables useful for evaluating patients with CAG, allowing for the recognition of those at increased risk of developing either epithelial GNL or T1gNET.

The epithelial GNL development was associated with low pepsinogen I levels and the presence of PCA. The low pepsinogen I level could be considered as the atrophy hallmark, as this serum biomarker is secreted by the chief cells of the oxyntic mucosa, which, in turn, is characterized by the reduction or disappearance of the canonical glands typically present in the gastric corpus, by definition in CAG patients. PCA define the autoimmune profile common in CAG, with PCA serving as a serological hallmark [2, 32, 33]. However, clinicians should be aware that PCA could decrease over time in CAG patients [34], and the mean age of seronegative CAG patients was significantly higher compared to PCA-positive patients. The same features, namely low pepsinogen I and PCA positivity, were also associated with the development of T1gNET. Although epithelial GNL and T1gNET are biologically distinct [1, 4, 24, 27], their coexistence in the predictive model likely reflects a shared pathogenic environment characterized by chronic inflammation and progressive corpus atrophy. In this context, these variables may act as markers of the inflammatory damage from which both lesions may arise.

Age over 60 years and antral inflammation, defined as mononuclear cell infiltration in the corresponding gastric region, are variables linked to GNL development, particularly epithelial GNL. In a retrospective single-center study, the development of GC/HG or low-grade dysplasia was associated with age over 60 years with a hazard ratio (HR) of 4.3 [7]. Antral inflammation is associated with epithelial GNL development. CAG with antral gastritis, atrophic or non-atrophic, is often linked to *H. pylori* infection [32], which may migrate from the antrum to the corpus over time [35, 36]. Studies indicate that *H. pylori* eradication can improve or regress mucosal injury [37, 38]. Therefore, the presence of antral inflammation could be associated with previous exposure to *H. pylori* infection, even though recent evidence suggests that antral inflammation may also be present in a subset of CAG patients and, in some cases, regress over time irrespective of *H. pylori* infection [39].

Low-dose aspirin therapy was associated with a lower likelihood of epithelial GNL development in our model, in line with previous observational studies reporting similar associations [40, 41]. A meta-analysis of 18 studies and a nationwide Korean cohort study (60,000 participants) reported lower GC risk in regular aspirin users over a median 4.7-year observation period [40]. However, specific analyses for low-dose aspirin use in CAG patients are lacking, preventing concrete recommendations.

The T1gNET development was associated with anti-thyroperoxidase antibodies, highlighting the well-known autoimmune comorbidity in CAG patients, such as autoimmune thyroiditis [2], defining a specific syndrome known as thyrogastric syndrome [42].

The SVM model identified an inverse association between *H. pylori* eradication therapy at the time of CAG diagnosis and the development of T1gNET. *H. pylori* infection is a well-known GC risk factor [43]. Specifically, patients with *H. pylori* infection, severe atrophic gastritis, corpus-predominant gastritis, or both, and intestinal metaplasia, are at higher risk for GC [44]. However, *H. pylori*-positive patients receiving eradication therapy at the baseline did not develop GC at follow-up. It is, therefore, evident that eradication therapy may represent one of the main strategies for preventing the development of GC [45], particularly in patients diagnosed with CAG. However, it should be acknowledged that in patients with gastric precancerous lesions, eradication therapy might not be effective in reversing intestinal metaplasia [46, 47].

The link between smoking, CAG, and GNL in corpus atrophy remains underexplored. However, a recent multicenter study [48] aimed at exploring risk factors associated with the development of gastric and duodenal neuroendocrine tumors revealed that smoking was more frequently reported in patients diagnosed with gastric neuroendocrine tumors compared to healthy controls. Specifically, in female patients, smoking was significantly associated with the development of gastric neuroendocrine tumors (OR 9.85, *p* = 0.001).

Dyspepsia is the final variable linked to T1gNET development and is a well-established symptom in CAG patients with gastrointestinal complaints [2, 32]. Achlorhydria, a common feature of CAG, may impair gastric motility and promote intestinal dysbiosis, contributing to symptoms [49]. In a study of CAG patients, 56.7% reported GI symptoms, with early satiety and postprandial fullness being the most frequent complaints [50]. Similarly, a multicenter study found CAG in 30.1% of patients undergoing upper GI endoscopy, identifying postprandial fullness as a clinical predictor for CAG [51]. These findings highlight the need to consider CAG in patients with persistent dyspepsia, as identifying symptoms like postprandial fullness could increase gastroscopy rates and may aid early GNL detection.

In the overall model evaluating the risk of developing GNL, without distinction between epithelial GNL and T1gNET, the variable hemoglobin was found to be inversely associated with GNL occurrence. This variable was selected while respecting its nature as a continuous variable, without defining specific reference thresholds—below which—the risk of GNL development would increase. This approach preserves the integrity of the dataset and aligns with the specific SVM model proposed. Lower hemoglobin levels, indicative of anemia, are clearly associated with an increased risk of GNL. Assessing anemia is a cornerstone in evaluating CAG patients, as both iron deficiency anemia and pernicious anemia are commonly observed in this condition and are well-documented in the literature [2, 32, 52]. Iron deficiency anemia can be the first hematological manifestation before the onset of pernicious anemia in 35–58% of patients with CAG [53]. In particular, pernicious anemia was associated with a higher risk of GNL development in CAG patients, displaying an HR of 4.3 and 2.2 in the development of epithelial GNL and T1gNET, respectively [7].

Our study has some limitations. The retrospective design could create an inclusion bias; however, the SVM system employed is specifically designed to minimize this bias. To achieve better performance, a larger dataset would be necessary to improve the model’s accuracy. However, the proposed SVM model was able to select, after appropriately handling missing data, feature subsets associated with the development of epithelial GNL and T1gNET, achieving sensitivity and specificity close to or greater than 80%. The overall number of GNL cases was limited, particularly T1gNETs, however, the study was conducted in a country with a low-to-intermediate risk for gastric cancer [54]. Our results may seem heterogeneous, and some may not be fully understood at the pathophysiological level. However, this is inherent in the development of AI systems, which use calculations and models that go beyond human learning. Therefore, the use of these systems should always be accompanied by medical expertise and knowledge for a comprehensive understanding of the pathology. It should also be noted that, as already stated in the Methods section, the term “corpus atrophic gastritis” (CAG) was preferred over “autoimmune gastritis” to ensure diagnostic clarity. This choice aimed to avoid misdiagnosis or misclassification, given the lack of universally accepted criteria for autoimmune gastritis [19]. Although the model was not externally validated using independent real-world cohorts, an additional validation step was performed using synthetic datasets specifically generated to reflect the structure and distribution of the original data. This approach allowed us to further assess the model’s generalizability, although prospective validation in independent datasets would be warranted in future studies.

In conclusion, this is the first study to propose an SVM model capable of identifying key variables associated with the development of GNL in patients with CAG and defining, from the time of diagnosis, the risk profile of such patients The specific characteristics of the model enable the selected variables to achieve strong sensitivity and specificity, when assessed collectively, evidencing the potential for AI-assisted systems to support clinicians in assessing the risk of developing GNL in patients with CAG and highlighting the importance of an integrated evaluation approach as outlined in this study.

## Supplementary Information

Below is the link to the electronic supplementary material.

Supplementary file1 (DOCX 14 KB)
