# Supplementary material for: Exploring the potential of artificial intelligence in assessing the risk of gastric neoplastic lesions in patients with corpus atrophic gastritis
Source: Gastric Cancer. 2025 Oct 22;29(1):159–68. doi: 10.1007/s10120-025-01679-7 (PMC12830466; doi:10.1007/s10120-025-01679-7)
Supplement: Supplementary file 1 — Supplementary file1 (DOCX 14 KB) [file 10120_2025_1679_MOESM1_ESM.docx]

**Supplementary Table 1:** Evaluation metrics for the quality assessment of the synthetic datasets.

|  | **SVC detection** | **Logistic detection** | **Category Coverage** | **Contingency Similarity** | **Discrete KL Divergence** | **Correlation Similarity** | **Statistic Similarity** |
| --- | --- | --- | --- | --- | --- | --- | --- |
| Epithelial lesion | 0.92 | 0.79 | 0.98 | 0.84 | 0.78 | 0.86 | 0.88 |
| T1gNET | 1.00 | 0.81 | 0.95 | 0.81 | 0.83 | 0.85 | 0.76 |
| No GNL | 0.99 | 0.91 | 1.00 | 0.91 | 0.89 | 0.93 | 0.97 |

**T1gNET:** type 1 gastric neuroendocrine tumor

**GNL:** gastric neoplastic lesion

**SVC:** Support Vector Classifier

**KL Divergence:** Kullback-Leibler divergence

**Supplementary Table 2:** Performance of the One‑Class SVM classifier on the synthetic test datasets

|  | **Se** | **Sp** |
| --- | --- | --- |
| Epithelial lesion | 92.3% | 86.4% |
| T1gNET | 88.9% | 92.6% |

**Se:** sensibility

**Sp:** specificity
